# Third‐line antiretroviral therapy, including raltegravir (RAL), darunavir (DRV/r) and/or etravirine (ETR), is well tolerated and achieves durable virologic suppression over 144 weeks in resource‐limited settings: ACTG A5288 strategy trial

**DOI:** 10.1002/jia2.25905

**Published:** 2022-06-15

**Authors:** Anchalee Avihingsanon, Michael D. Hughes, Robert Salata, Catherine Godfrey, Caitlyn McCarthy, Peter Mugyenyi, Evelyn Hogg, Robert Gross, Sandra W. Cardoso, Aggrey Bukuru, Mumbi Makanga, Sharlaa Badal‐aesen, Vidya Mave, Beatrice Wangari Ndege, Sandy Nerette Fontain, Wadzanai Samaneka, Rode Secours, Marije Van Schalkwyk, Rosie Mngqibisa, Lerato Mohapi, Javier Valencia, Patcharaphan Sugandhavesa, Esmelda Montalban, Cornelius Munyanga, Maganizo Chagomerana, Breno R. Santos, Nagalingeswaran Kumarasamy, Cecilia Kanyama, Robert T. Schooley, John W. Mellors, Carole L. Wallis, Ann C. Collier, Beatriz Grinsztejn, for the A5288 Study team

**Affiliations:** ^1^ HIV‐NAT, Thai Red Cross AIDS Research Centre and Centre of Excellence in Tuberculosis Faculty of Medicine Chulalongkorn University Bangkok Thailand; ^2^ Center for Biostatistics in AIDS Research in the Department of Biostatistics Harvard T H Chan School of Public Health Boston Massachusetts USA; ^3^ Case Western Reserve University Cleveland Ohio USA; ^4^ Division of AIDS National Institutes of Allergy and Infectious Disease National Institutes of Health Bethesda Maryland USA; ^5^ Joint Clinical Research Center Kampala Uganda; ^6^ Social & Scientific Systems Inc. a DLH Holdings Company Silver Spring Maryland USA; ^7^ Center for Clinical Epidemiology and Biostatistics University of Pennsylvania Philadelphia Pennsylvania USA; ^8^ Instituto Nacional de Infectologia Evandro Chagas Fundacao Oswaldo Cruz Rio de Janeiro Brazil; ^9^ Kenya Medical Research Institute/Center of Disease Control Kisumu Kenya; ^10^ Clinical HIV Research Unit Helen Joseph Hospital University of Witwatersrand Johannesburg South Africa; ^11^ BJ Medical College Clinical Research Site Pune India; ^12^ Moi University Clinical Research Center (MUCRC) CRS Eldoret Kenya; ^13^ Les Centres GHESKIO Clinical Research Site Port‐au‐Prince Haiti; ^14^ University of Zimbabwe Clinical Trials Research Centre Harare Zimbabwe; ^15^ Family Centre for Research with Ubuntu (FAMCRU) Stellenbosch University Cape Town South Africa; ^16^ Durban International Clinical Research Site, King Edward Hospital, Enhancing Care Foundation Durban South Africa; ^17^ Soweto AIDS Clinical Trials Group Clinical Research Site, Perinatal HIV Research Unit University of the Witwatersrand Johannesburg South Africa; ^18^ Barranco Clinical Research Site Lima Peru; ^19^ Research Institute for Health Sciences Chiang Mai University Chiang Mai Thailand; ^20^ San Miguel Clinical Research Site Lima Peru; ^21^ University of North Carolina Project, Kamazu Central Hospital Lilongwe Malawi; ^22^ Hospital Nossa Senhora da Conceicao CRS Porto Alegre Brazil; ^23^ CART Clinical Research Site VHS Infection Disease Medical Centre Chennai India; ^24^ Division of Infectious Diseases University of California San Diego California USA; ^25^ Division of Infectious Diseases Department of Medicine University of Pittsburgh School of Medicine Pittsburgh Pennsylvania USA; ^26^ BARC‐South Africa and Lancet Laboratories Johannesburg South Africa; ^27^ University of Washington School of Medicine University of Washington Seattle Washington USA

**Keywords:** A5288, darunavir, drug resistance, LMIC, third‐line ART, 144 weeks efficacy

## Abstract

**Introduction:**

ACTG A5288 was a strategy trial conducted in diverse populations from multiple continents of people living with HIV (PLWH) failing second‐line protease inhibitor (PI)‐based antiretroviral therapy (ART) from 10 low‐ and middle‐income countries (LMICs). Participants resistant to lopinavir (LPV) and/or multiple nucleotide reverse transcriptase inhibitors started on third‐line regimens that included raltegravir (RAL), darunavir/ritonavir (DRV/r) and/or etravirine (ETR) according to their resistance profiles. At 48 weeks, 87% of these participants achieved HIV‐1 RNA ≤200 copies/ml. We report here long‐term outcomes over 144 weeks.

**Methods:**

Study participants were enrolled from 2013 to 2015, prior to the availability of dolutegravir in LMICs. “Extended Follow‐up” of the study started after the last participant enrolled had reached 48 weeks and included participants still on antiretroviral (ARV) regimens containing RAL, DRV/r and/or ETR at that time. RAL, DRV/r and ETR were provided for an additional 96 weeks (giving total follow‐up of ≥144 weeks), with HIV‐1 RNA measured at 48 and 96 weeks and CD4 count at 96 weeks after entry into Extended Follow‐up. Proportion of participants with HIV‐1 RNA ≤200 copies/ml was estimated every 24 weeks, using imputation if necessary to handle the different measurement schedule in Extended Follow‐up; mean CD4 count changes were estimated using loess regression.

**Results and Discussion:**

Of 257 participants (38% females), at study entry, median CD4 count was 179 cells/mm^3^, and HIV‐1 RNA was 4.6 log_10_ copies/ml. Median follow‐up was 168 weeks (IQR: 156–204); 15 (6%) participants were lost to follow‐up and 9 (4%) died. 27/246 (11%), 26/246 (11%) and 13/92 (14%) of participants who started RAL, DRV/r and ETR, respectively, discontinued these drugs; only three due to adverse events. 87%, 86%, 83% and 80% of the participants had HIV‐1 RNA ≤200 copies/ml at weeks 48, 96, 144 and 168 (95% CI at week 168: 74–85%), respectively. Mean increase from study entry in CD4 count at week 168 was 265 cells/mm^3^ (95% CI 247–283).

**Conclusions:**

Third‐line regimens comprising of RAL, DRV/r and/or ETR were very well tolerated and had high rates of durable virologic suppression among PLWH in LMICs who were failing on second‐line PI‐based ART prior to the availability of dolutegravir.

## 1 INTRODUCTION

Mathematical modelling suggests that by 2030, up to 4.6 million people living with HIV (PLWH) will require second‐line antiretroviral therapy (ART) globally [1]. Studies in Asia and Africa have reported second‐line treatment failure rates of 8–40% [2, 3, 4, 5]. Ritonavir‐boosted lopinavir (LPV/r) was recommended for second‐line protease inhibitor (PI)‐based ART in low‐ and middle‐income countries (LMICs), but adherence was difficult due to intolerability and high pill burden. Treatment of PLWH with viremia on second‐line ART can be challenging in LMICs due to uncertainty about resistance and limited data on virologic response to other regimens. Some PLWH needing third‐line therapy may have been exposed to a variety of nucleoside/nucleotide reverse transcriptase inhibitors (NRTIs), non‐nucleoside reverse transcriptase inhibitors (NNRTIs) and PIs, and their drug‐resistance patterns are variable and complex, particularly as treatment failure on PI‐based ART may involve multiple PI mutations [6]. A systematic review found that only two‐thirds of patients receiving PI‐based second‐line ART in sub‐Saharan Africa achieved virologic suppression [7], suggesting adherence challenges. After prolonged exposure to failing ART, accumulation of drug resistance is unavoidable [8] and reduces future treatment options. Ritonavir‐boosted darunavir (DRV/r), integrase strand transfer inhibitors (INSTIs) and etravirine (ETR) were recommended by the WHO as third‐line ART in 2016 [9]. With limited HIV‐1 RNA and resistance testing in many LMICs, durable potent and tolerable ART is critical. There are, however, limited data about long‐term outcomes of third‐line ART in LMICs.

To address this gap, the ACTG A5288 study enrolled participants at 19 urban sites in LMICs in Africa (Kenya, Malawi, South Africa, Uganda and Zimbabwe), Latin America (Brazil, Haiti and Peru) and Asia (India and Thailand) during 2013–2015, prior to the availability of dolutegravir (DTG). A5288 included evaluation of third‐line regimens containing DRV/r plus raltegravir (RAL) with either ETR or two NRTIs in PLWH experiencing virologic failure (VF) on their PI‐based second‐line regimen with PI resistance and/or resistance to multiple NRTIs. In primary results, 87% of participants on these regimens achieved HIV‐1 RNA ≤200 copies/ml at week 48 [14]. Here, we report long‐term outcomes over a median of 168 weeks in this diverse population of PLWH who used DRV/r+RAL+/–ETR as third‐line ART.

## 2 METHODS

### 2.1 Study design and participants

The design of ACTG A5288 and primary results at week 48 have been published [14]. In brief, participants were assigned to one of four cohorts based on real‐time drug resistance results and treatment history. Cohort A (no LPV resistance and susceptible to at least one NRTI) stayed on their second‐line ART regimen (this cohort is excluded from this report). Participants with LPV/r resistance and/or resistance to NRTIs were assigned to cohorts B or C, which prescribed regimens, including DRV/r and RAL, with either ETR or optimized NRTIs. In cohort D, for participants with the most complex resistance profile, the best regimen was constructed using study‐provided or locally available agents.

**Figure 1:**
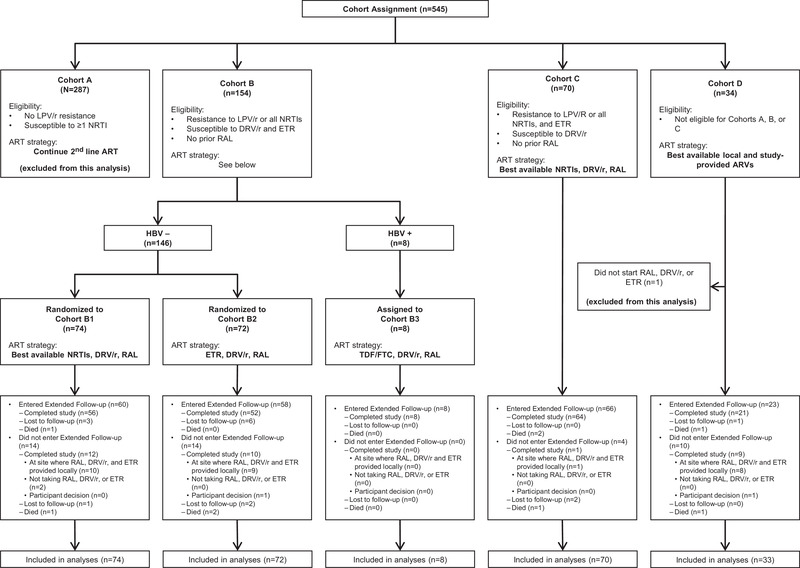
Cohort definitions, assignment, treatment strategy and follow‐up. Abbreviations: DRV, darunavir; ETR, etravirine; FTC, emtricitabine; RAL, raltegravir; RTV, ritonavir; 3TC, lamivudine; TDF, tenofovir disoproxil fumarate.

For participants experiencing VF, another resistance test was performed with the possibility of changing treatment based on resistance results, similar to the process performed at study entry (Figure 1). All participants were initially followed until the last participant reached 48 weeks. Participants still taking RAL, DRV/r or ETR who were at sites where these drugs were not locally available were then eligible to enter “Extended Follow‐up;” these participants were followed every 24 weeks for a further 96 weeks with HIV‐1 RNA measured at 48 and 96 weeks and CD4 count at 96 weeks.

This study was approved by site‐specific ethics committees. All participants gave their written informed consent.

### 2.2 Statistical analysis considerations

This report describes long‐term outcomes of 257 study participants who initially received one or more of RAL, DRV/r and ETR in cohorts B, C and D (Figure 1; one cohort D participant did not start any of these drugs and was excluded). Treatment discontinuation at any time during follow‐up was defined as permanent discontinuation of any drug in the regimen initially started in the study (except changes due to local drug availability).

Suppression of HIV‐1 RNA ≤200 copies/ml was evaluated every 24 weeks, with imputation if needed because of the reduced measurement schedule in Extended Follow‐up: HIV‐1 RNA was imputed as ≤200 copies/ml if both the preceding and succeeding measurements were ≤200 copies/ml and, otherwise, imputed as >200 copies/ml (including due to death or loss to follow‐up). Changes in CD4 count were estimated using loess regression.

## 3 RESULTS AND DISCUSSION

Characteristics at study entry are presented in Table 1. Median CD4 count was 179 cells/mm^3^, HIV‐1 RNA was 4.6 log_10_ copies/ml and 38% were females. Median time on ART prior to study entry was 8.0 years. All participants in cohorts B and C and 48% of participants in cohort D showed DRV susceptibility.

**Table 1:** Characteristics of the cohort, including drug resistance at study entry

	B1 (*N* = 74)	B2 (*N* = 72)	B3 (*N* = 8)	C (*N* = 70)	D (*N* = 33)	Total (*N* = 257)
Age, years						
Median (IQR)	41 (34, 49)	43 (36, 48)	42 (34, 45)	42(35, 46)	43 (38, 48)	42 (36, 47)
Sex, *n* (%)						
Female	29 (39%)	28 (39%)	4 (50%)	23 (33%)	14 (42%)	98 (38%)
Region, *n* (%)						
Africa	40 (54%)	41 (57%)	4 (50%)	32 (46%)	17(52%)	134 (52%)
Asia	21 (28%)	17 (24%)	3 (38%)	35 (50%)	8 (24%)	84 (33%)
South America	10 (13%)	9 (13%)	0 (0%)	1 (1%)	8 (24%)	28 (11%)
Caribbean	3 (4%)	5 (7%)	1 (13%)	2 (3%)	0 (0%)	11 (4%)
Screening plasma HIV‐1 RNA, log_10_ copies/ml						
Median (IQR)	4.6 (3.7, 5.2)	4.6 (3.7, 5.4)	3.9 (3.2, 4.8)	4.6 (3.7, 5.4)	4.2 (3.7, 5.1)	4.6 (3.7, 5.3)
% >100,000 copies/ml	31%	33%	25%	41%	39%	35%
CD4 count, cells/mm^3^						
Median (IQR)	174 (50, 317)	198 (71, 314)	250 (197, 322)	161 (71, 289)	173 (62, 361)	179 (68, 313)
% <50 cells/mm^3^	23%	15%	13%	13%	21%	18%
Time on ART, years						
Median (IQR)	8.3 (5.9, 11.7)	7.9 (6.1, 10.0)	7.4 (5.8, 11.1)	8.1 (6.2, 9.9)	7.9 (5.8, 10.1)	8.0 (6.1, 10.6)
Drug resistance, %						
NRTI	95%	90%	100%	96%	91%	93%
NNRTI^a^	74%	76%	75%	99%	79%	82%
PI	88%	83%	88%	79%	91%	84%
LPV/r susceptible	20%	24%	25%	33%	12%	
DRV susceptible	100%	100%	100%	100%	48%	
ETR susceptible	100%	100%	100%	3%^b^	67%	

Abbreviations: ART, antiretroviral therapy; DRV, darunavir; ETR, etravirine; IQR, interquartile range; LPV/r, lopinavir/ritonavir; NNRTI, non‐nucleoside reverse transcriptase inhibitor; NRTI, nucleoside reverse transcriptase inhibitor; PI, protease inhibitor.

^a^ NNRTI resistance refers to any level of resistance to NVP, EFV and ETR.

^b^ These participants showed the evidence of ETR resistance in a historical genotype but not in the screening genotype.

Overall, 233 (91%) completed study follow‐up, 9 (4%) died and 15 (6%) were lost to follow‐up. Median follow‐up (including Extended Follow‐up) was 168 weeks. Thirty‐five participants (14%) permanently discontinued one or more drugs in the regimen started in the study. Reasons for discontinuation were death (4%), adverse events (3%), loss to follow‐up (2%), non‐compliance (2%), VF with new resistance mutations (1%) and other reasons (1%). Of participants who started RAL, DRV/r and ETR, 27/246 (11%), 26/246 (11%) and 13/92 (14%), respectively, discontinued these drugs; only three due to adverse events (rash and skin discolouration, increased alkaline phosphatase and increased bilirubin). Only three participants (one in cohort B2 and two in cohort D) discontinued any of these drugs due to resistance. The one in cohort B2 developed INSTI resistance in Extended Follow‐up identified on a resistance test obtained outside of the study, discontinued RAL, DRV/r and ETR, and was subsequently discontinued from the study. Of the two in cohort D, one developed the Y181C mutation and changed from ETR to RAL and one developed the E92Q and N155H mutations and changed from RAL to DRV/r.

Any grade serious adverse event, Grade≥3 signs and symptoms, Grade≥3 laboratory abnormalities and Grade≥3 diagnoses occurred in 23%, 15%, 29% and 23% of participants, respectively. Clinical events included AIDS‐defining events (7%), non‐AIDS‐defining events (14%), hospitalizations (16%) and pregnancies (4%).

**Figure 2:**
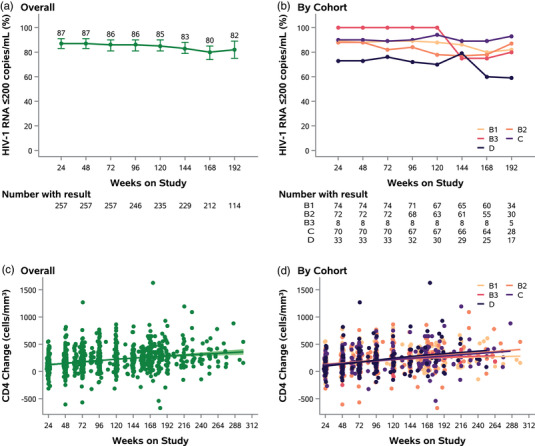
HIV‐1 RNA and CD4^+^ count outcomes. Note: Shown in Panels 2a and b are the percentages of participants with HIV‐1 RNA ≤200 copies/ml at every 24 weeks during study follow‐up, both overall (Panel 2a) and by cohort (Panel 2b). The vertical lines around the data points in Panel 2a represent Wald 95% confidence intervals. The points shown in Panels 2c and d are changes in CD4^+^ count from study entry for all available measurements during study follow‐up, both overall (Panel 2c) and by cohort (Panel 2d). Trend lines represent non‐parametric locally weighted regression (locally estimated scatterplot smoothing [loess]) lines. The band in Panel 2c represents the 95% confidence interval for the trend line. For visual clarity, confidence intervals were omitted from Panels 2b and d.

An estimated 87%, 86%, 83% and 80% of participants had HIV‐1 RNA ≤200 copies/ml at weeks 48, 96, 144 and 168 (95% CI at week 168: 74–85%), respectively (Figure 2a and b). Among 29 participants with observed HIV‐1 RNA >200 copies/ml at week 48 (so excluding five participants who died or were lost to follow‐up before week 48), 19 had a result at week 144 (the remaining 10 were not followed to week 144). Among these 19 participants, 13 (68%) were ≤200 copies/ml at week 144 measurement.

Cohort D, which had the most extensive resistance, generally had the lowest proportion of participants with HIV‐1 RNA suppressed throughout follow‐up. The two randomized cohorts (cohort B1, which received best available NRTIs, DRV/r and RAL, and cohort B2, which received ETR, DRV/r and RAL) had similar suppression rates: 88% in both cohorts at week 48, and 80% versus 78% at week 168.

There was a gradual increase in CD4 count over time in all cohorts (Figure 2c and d): mean CD4 count was 150, 201, 245 and 265 (95% CI 247–283) cells/mm^3^ at weeks 48, 96, 144 and 168, respectively.

## 4 CONCLUSIONS

In LMICs where frequent HIV RNA testing is not accessible, access to antiretroviral (ARV) regimens with robust efficacy and durable HIV RNA suppression is critical. Although DTG is currently recommended globally for both first‐line and second‐line therapies after failure of non‐DTG‐containing first‐line regimens, our results remain relevant for PLWH who have experienced treatment failure on both NNRTI‐ and PI‐based regimens. A5288 is an important trial in LMICs of treatment options for PLWH of various ethnicities, cultures and socio‐economic backgrounds failing second‐line PI‐based regimens with PI resistance and/or resistance to multiple NRTIs, which used real‐time genotyping testing to select third‐line regimens using an algorithmic approach [10]. We found sustained high rates of virologic suppression (over 80%) and increased CD4 counts, and low rates of clinical events and treatment‐limiting adverse events among participants taking third‐line regimens, including DRV/r, RAL and/or ETR over a median of 168 weeks of follow‐up. Consistent with the findings of EARNEST [11] and NADIA [12] for second‐line ART in Africa, our results show that NRTIs, particularly TDF/FTC or TDF/3TC, can be effectively recycled with highly efficacious third‐line drugs, such as DRV/r.

These results are of critical importance in showing that, if highly tolerable suppressive ART is available, virologic suppression can be achieved even after sequential treatment failures, with beneficial results not only for delaying HIV progression but also in preventing onward HIV transmission. The high proportion of participants achieving long‐term virologic suppression in this study is in line with that seen in observational studies in LMICs. In South Africa, 82.9% of PLWH on salvage ARV regimens achieved HIV‐1 RNA <400 copies/ml with median follow‐up of 2.5 years [13]. Zimbabwe's third‐line ART program reported 90% suppression (<200 copies/ml) with median follow‐up of 1.4 years [14]. However, reports from the South African public health sector showed only 58% virologic suppression [15]. The Thilao study, in West Africa, found low (59%) HIV‐1 RNA suppression at week 64, even though participants were given intensive support for treatment adherence and HIV‐1 RNA testing was conducted more frequently than in clinical practice [16]. Nevertheless, in our cohort and other cohorts mentioned above, 10–40% had HIV‐1 RNA >200 copies/ml. For PLWH who have difficulty maintaining good adherence on oral regimens, injectable long‐acting ART, such as cabotegravir‐rilpivirine or lenacapavir, may be helpful.

Our findings are particularly important because data regarding long‐term outcomes after more than 3 years of third‐line ART in LMICs are scarce. In Extended Follow‐up, HIV‐1 RNA was only monitored annually, reflecting clinical practice in many LMICs. Therefore, the robust durability and tolerability of our third‐line regimens are likely generalizable to these settings. However, some limitations should be acknowledged. First, RAL, which was used in this study because of its availability when the study was initiated, is more expensive than generic DTG and requires twice daily rather than once daily dosing. Second, we did not have data on drug concentrations and genotypic drug‐resistance testing at VF in Extended Follow‐up, so we cannot evaluate whether the VF was due to poor adherence, or emergence of new mutations. However, the majority (63%) of participants with HIV‐1 RNA >200 copies/ml at week 48 were re‐suppressed to <200 copies at week 144 suggesting adherence issues.

In conclusion, among PLWH from LMICs who failed NNRTI‐ and PI‐based regimens, third‐line regimens containing RAL, DRV/r and/or ETR were well tolerated and provided a high rate of durable virological suppression over 3 years.

## COMPETING INTERESTS

ACC has received a research grant from Bristol‐Myers Squibb and honoraria from Merck & Co. for Data Monitoring Committee membership. JWM is a consultant to and grant recipient from Gilead Sciences and owns shares in Abound Bio (unrelated to the current work) and has received share options in Infectious Disease Connect (unrelated to the current work). All other authors declare no competing interests.

## AUTHORS’ CONTRIBUTIONS

AA contributed to the literature search, participant recruitment, data collection and interpretation, and manuscript drafting and revision. MDH contributed to the study design, data analysis and interpretation, and manuscript drafting and revision. CM contributed to the figures, data analysis and interpretation, and manuscript drafting and revision. RS, EH, CG and RTS contributed to the study design, data interpretation and manuscript revision. PM contributed to the study design, participant recruitment and data collection, and manuscript revision. SWC, AB, MM, SBF, VM, BWN, SNF, WS, RS, MvS, RM, LM, JV, PS, EM, CM, MC, BRS, NK and CK contributed to participant recruitment, data collection and interpretation, and manuscript revision. JWM contributed to the study design, virological resistance studies, data interpretation and manuscript revision. CLW contributed to the literature search, study design, virological resistance studies, data interpretation, and manuscript drafting and revision. ACC contributed to the literature search, study design, data interpretation and manuscript revision. BG contributed to the literature search, study design, participant recruitment, data collection and interpretation, and manuscript revision.

## FUNDING

Research reported in this publication was supported by the National Institutes of Allergy and Infectious Diseases of the National Institutes of Health under Award numbers (UM1 AI068636, UM1 AI068634 [ACTG Statistical and Data Management Center], UM1 AI069423 and UM1 AI069481).

## ROLE OF THE FUNDING SOURCE

The United States’ National Institutes of Health were the study funders and had an oversight role in the development and monitoring of the study. One author (CG) was an employee of this sponsor and a member of the study team involved in the conduct, analyses and interpretation/conclusions of the study. The corresponding author had full access to all the study data and had final responsibility for the decision to submit for publication.

## DISCLAIMER

The content is solely the responsibility of the authors and does not necessarily represent the official views of the National Institutes of Allergy and Infectious Diseases. AbbVie, Gilead Sciences, Janssen Pharmaceuticals and Merck & Company provided the study drugs.

## Data Availability

The authors confirm that all data underlying the findings are fully available upon request from sdac.data@sdac.harvard.edu with the written agreement of the AIDS Clinical Trials Group.
